# From fixer to facilitator: an interpretative phenomenological study of diabetes person-centred counselling and empowerment education

**DOI:** 10.12688/f1000research.73596.1

**Published:** 2022-01-21

**Authors:** Florence Findlay-White, Tim Dornan, Mark Davies, Alan Archer, Anne Kilvert, Charles Fox

**Affiliations:** 1 Centre for Medical Education, Queen's University Belfast, Belfast, Northern Ireland, BT9 7BL, UK; 2 Clinical Psychology Department, Belfast City Hospital, Belfast, Northern Ireland, BT9 7AE, UK; 3 Derbyshire Community Health Service, Walton Hospital, Chesterfield, England, S40 3HW, UK; 4 Northampton Community Diabetes Service, Danetre Hospital, Daventry, England, NN11 4DY, UK; 5 Diabetes Research Centre, University of Leicester, Leicester, England, LE5 4PW, UK

**Keywords:** Person-centred approach, health professional education, counselling, facilitation, qualitative research, empowerment education, diabetes

## Abstract

**Background:** The purpose of this study is to explore the professional and personal experiences of multidisciplinary healthcare professionals during and following diabetes counselling and empowerment education.

**Methods:** Everyone who had participated in a diabetes counselling and empowerment course between 2008-2016 was invited to respond to an online survey and follow-up telephone interview if willing. Interviews were recorded and transcribed verbatim. The research team used interpretative phenomenology to identify core themes from both the survey and telephone interviews and which captured the impact of empowerment education.

**Results:** 22 doctors, nurses, dieticians, and psychologists completed an online questionnaire. 10 subsequently took part in telephone interviews. Empowerment education changed them from fixers to facilitators. Their transformation included a sense of becoming authentic, ‘being the way I want to be’ in clinical practice and becoming more self-reflective. This affected them personally as well as reinvigorating them professionally.

**Conclusions:**The participants described a personal and professional journey of transformation that included discovering their person-centred philosophy. They adopted a consultation structure that empowered people with diabetes to care for themselves. It can be speculated that participants’ experience of transformation may also guard against professional burnout.

## Introduction

Diabetes care focuses on blood glucose management to prevent micro and macro vascular complications. Success depends mainly on individual self-management, including dietary behaviours, medication/insulin management, and monitoring. The ability to self-manage is influenced by psychosocial aspects, which present wide-ranging and individual barriers. Living with diabetes can impose a psychological burden, including a higher prevalence of distress and depression, which predict poor physical outcomes. ^1^^,^^2^ People with diabetes have asked professionals to pay attention to their psychological health. ^3^^,^^4^ This requires professionals, many of whom were trained in the traditional medical paradigm, to provide care that supports self-management. ^5^ The remit of the professional is to support behaviour change, but this can only be effective when framed in the context of the person’s life. ^6^

Many educators have developed courses to teach psychosocial care, but evidence of effectiveness is limited. Courses most often concentrate on communication skills, for which there is some evidence of effectiveness, mainly in cancer care and primary care. ‘High-intensity’ courses (lasting days rather than hours) delivered by clinicians or researchers with curricula that include cognitive, behavioural, and affective skills development, filmed role play, and individualised feedback have helped clinicians elicit people’s’ concerns. ^7^ These have also helped clinicians exhibit a person-centred consultation style including expressing empathy. ^8^^,^^9^ Transfer of these skills into everyday practice cannot be assumed. ^10^^,^^11^ There is a dearth of well-evaluated interventions that more directly target behaviour change, extend beyond communication skills, and examine longer-term impact.

The diabetes five-step empowerment model was first introduced in 1991. ^12^ Patients identify their issues/problems, explore their thoughts and feelings in relation to these, consider options, make a plan for change and finally how to evaluate the plan.

Successful empowerment education requires a person-centred collaborative relationship between clinician and the person with diabetes. This can be achieved when the clinician is able to let go of the traditional medical model of socialisation towards themself as expert and the person with diabetes as passive, which is also reinforced by workplace hierarchies. ^13^ This paradigm shift ^14^ requires skills and a consultation structure (the empowerment model) that enables the clinician to help people to reflect on their lives with diabetes and decide what is important to them and what they wish to change.

The empowerment approach focuses on the experience of life with diabetes including eliciting barriers to diabetes self-management, exploring the burden of living with diabetes, and facilitating patient identified goals and planning for change. ^12^

One way the empowerment approach has been put into practice in the UK is through a three-day course in which participants develop a philosophy based on the Person-Centred Approach to counselling created by Carl Rogers. ^15^ In 2008, Professor Bob Anderson from the University of Michigan met with the faculty of an existing UK counselling course to help redesign it to include the empowerment model of education. This has enabled participants to combine the person-centred approach with the five-step empowerment model within a consultation structure. ^16^

The present research results from over a decade’s experience of using this approach to train healthcare professionals to help people with diabetes optimise their diabetes self-management.

Course participants said that attending the course not only enhanced their care for people with diabetes but had a ‘transformative’ effect on their clinical practice and their personal lives. This study focuses on the experience of the participants during and following training in person-centred empowerment education. Reasoning that a clearer understanding of participants’ experiences might provide much-needed insight into the education of healthcare professionals for psychosocial care, the authors set out to explore the phenomenon.

## Methods

### Ethics approval

Ethical approval was obtained from the School of Medicine, Dentistry and Biomedical Sciences Research Ethics Committee, Queen’s University, Belfast.

### Study design

This was an in-depth, exploratory qualitative analysis of the experiences of an opportunity sample of health professionals. It was designed to clarify professionals’ lived experiences of participating in an educational intervention grounded in person-centred philosophy and the diabetes empowerment approach.

### Conceptual orientation

Qualitative research is constructivist in the sense that researchers engage subjectively with what participants say, and ‘construct’ interpretation. Whilst this does not ‘prove’ that relationships exist, it does provide rich descriptions of social phenomena, which can be transferred to other people in other places. Rigour is enhanced by giving qualitative research projects an explicit theoretical orientation, linking the findings to a wider body of knowledge, rather than researchers’ whim. The methodology of this project was interpretative phenomenology, which interprets people’s accounts of their life experiences. The researcher makes sense of, or interprets, participants’ experiences within the context of the study. ^17^^,^^18^

### The intervention

This research treats the empowerment course as a ‘complex intervention’, whose impact can be explored by examining participants’ subjective experiences. The curriculum is designed to enable delegates to reflect on what person-centred care means to them. The course is held over three days, twice a year, in England and Ireland. The venues are residential and relatively remote, which helps participants reflect without outside distractions. Each course is made up of 18 participants and six trained multidisciplinary healthcare facilitators. These include psychologists, a humanistic person-centred therapist, diabetes specialist dietitian and nurses, and consultant diabetologists.

Participants are introduced to person-centred theory including understanding the core conditions of person-centred practice. ^19^ They practise a range of skills that communicate person-centred care using video real play in small groups made up of three participants and one facilitator. Within the groups, each participant takes their turn to experience the roles of counsellor, client, and observer. When taking the client role, participants are asked to bring an issue that is real to them (rather than roleplay) as this enhances learning through self-awareness and reflection. Feedback sessions within the small groups are led by the counsellor, using interpersonal process recall. ^20^ This allows learning through reflection in a confidential, unthreatening environment. At the end of the course, trained actors role-play people with diabetes enabling delegates to practice what they have learned in a work-related context. Table 1 shows a course programme outlining the components and structure of the course.

**Table 1.** Course programme.

Programme
• Arrival Time from 2 pm for Check-In for 4 pm start as below
• Because of the nature of the course, these times are flexible
LG = Large Group
SG = Small Group
3 participants and a facilitator
TUESDAY Welcome and what do I want to achieve?
16.00 Introductions • Orientation to course content • Course philosophy: qualities, principles, core conditions • ‘Person centred collaborative care‘
19.00 Dinner
20.00 Giving the Right Messages
WEDNESDAY Problem Exploration
09.00 Revisiting counselling principles: core conditions and person centred collaboration • Introduction to empowerment model: stages 1 & 2
09.45 Preparation for first ‘real play‘ • Video demo #1 & 2 – problem exploration and identification of feelings
10.30 1st Real Play (5 mins + 5 mins skills feedback)
11.15 Coffee
11.45 Interpersonal process recall (IPR) demonstration
12.30 Skills feedback and IPR
13.15 Lunch
14.15 What exactly were the counsellors doing or saying that worked well? • Identification of skills
14.45 2nd Real Play (10 mins + 10 min skills feedback)
15.45 Tea
16.15 Skills feedback and IPR
17.30 Reflective round • What are you taking away from today’s experiences?
18.00 End of session
THURSDAY Goal Setting & Action Planning
09.00 Empowerment stage 3 Goal setting LG • Demo video #3
09.45 3rd Real Play SG
10.30 Coffee
11.00 Empowerment stage 4. Action Planning • Demo video #4
11.00 Empowerment stage 4. Action Planning • Demo video #4 11.45 4th Real Play SG
13.00 Lunch
14.00 Continue 4th Real Play + IPR SG
15.00 • What are the barriers that get in the way of this process? • The personal experience of learning new skills LG
15.45 Tea
16.15 Final Real Play session SG
17.15 Actor preparation • Reflective round LG • What are you taking away from today?
18.00 Close
FRIDAY
09.00 Organisation of actor groups LG
09.15 Actor roleplay x 3 for each group SG
11.00 Coffee
11.30 • Application in the real world LG • Course evaluation
12.30 Final reflective round and goodbye
13.00 End of course (Lunch)

### Research team and their reflexive involvement

Five of the authors who are also course facilitators (FF-W, AK, AA, CF and MD) wrote autoethnographies of 200-400 words reflecting on their own experiences of the course, and their views on the topics of empowerment and diabetes counselling. This step helped them be reflexive; in other words, participate in the research, whilst remaining conscious of their own subjective positions. ^21^ FFW, a female researcher and diabetes specialist nurse who has an MPhil in phenomenological research led the study and interviewed participants. She was well known to participants as a course facilitator and, more widely, a national leader in person-centred diabetes care. The other four authors supported her role in the research by helping her be aware of her presuppositions about the topic, conduct interviews impartially, and interpret participants’ responses with very well-informed and yet detached curiosity about what they said. Technically, this is known as adopting ‘the phenomenological attitude’.

### Recruitment and participants

125 multidisciplinary healthcare professional who attended courses between 2008-2016 and whose email addresses were available were informed about the project and invited to complete an online survey (Letter to Participants in *Extended data*).

The online survey comprised four demographic questions and four open-ended questions about their experiences during and after the course (Online Survey Questions in *Extended data*). Some contact details held for participants who had attended the course in earlier years were likely to be no longer in use, so our sampling strategy was opportunistic which is in keeping with the relativist (all views are equally valid) framework of the research.

Response to the questionnaire was taken as informed consent to take part in the study. Attendance at the course was the only inclusion criterion. Unwillingness to participate was the only exclusion.

Participants willing to take part in telephone interviews gave contact details at the end of the questionnaire. They were sent a separate invitational email for a telephone interview and informed consent was agreed by response to email.

### Data collection

Telephone interviews focused on participants’ personal experiences during and after completing the course. In accordance with phenomenological methodology, questions were open-ended and minimally structured to elicit the unique experiences of each participant (Interview Guide in *Extended data*). Interviews were carried out during 2017. Length of interview was determined by the depth in which a participant was willing or able to discuss the topic. Each interview was recorded and transcribed verbatim.

### Analytical procedures

The dataset comprised written responses to the online survey and telephone interviews. To preserve confidentiality and avoid bias, the lead researcher (FFW) withheld the names of interviewees and pseudonymized the transcripts. All researchers listened to the audio-recordings as well as reading the transcripts. FFW used the Template Analysis method developed specifically to manage phenomenological research data. She started by systematically populating the Template with a priori themes drawn from researchers’ autoethnographies. ^22^ She then used participants’ responses to the open-ended questions in the online survey to expand those themes. Next, she read individual telephone interviews closely, creating a progressively more sophisticated set of codes and organising these into higher-level interpretative themes, which informed the final written report of findings. Throughout this, she explored how participants experienced person-centred empowerment education, consciously doing so from a phenomenological stance. Co-researchers helped her do this by discussing her interpretation and how preconceptions and biases might have influenced this. We draw heavily on the participants’ own words to report the findings using pseudonyms to disguise the identities of participants.

## Results

### Demographic results

37 participants (27%) responded to the online survey of which 22 were fully completed ( Table 2).

**Table 2.** Demographics.

Pseudonyms	Gender	Professional discipline	Years in practice	Year participated on course
Interviewed				
Pauline	F	Diabetes specialist nurse	21-30	2016
James	M	GP	21-30	2015
Nadia	F	Consultant physician	10-20	2015
Heather	F	Psychologist	21-30	2016
Sue	F	Diabetes specialist nurse	31-40	2015
Naveed	M	Specialty registrar doctor	0-10	2016
Jenny	F	Paediatric diabetes specialist nurse	21-30	2015
Becky	F	Diabetes specialist nurse	21-30	2016
Mary	F	Dietician	21-30	2015
Fran	F	Diabetes specialist nurse	31-40	2016
Survey only				
David	M	Psychologist	10-20	2015
Patsy	F	Specialty registrar doctor	10-20	2013
Rudi	F	Paediatric diabetes consultant	21-30	2012
Sarah	F	Diabetes specialist nurse	21-30	2010
Leanne	F	Diabetes specialist nurse	21-30	2016
Josh	M	Consultant physician	10-20	2008
Kate	F	Diabetes specialist nurse	10-20	2012
Iris	F	Diabetes specialist nurse	10-20	2013
Ben	M	Specialty registrar doctor	0-10	2012
Jonathan	M	Diabetes specialist nurse	10-20	2015
Paula	F	Practice nurse	0-10	2015
Aisling	F	Consultant physician	10-20	2015
Beverley	F	Practice nurse	21-30	2016

16 (73%) of the 22 responders to the online survey were women, reflecting the gender ratio of the course participants. Most were nurses or doctors with at least 11 years of professional experience. 15 (68%) participated in the 2015 and 2016 courses. 10 responders (5 nurses, 3 doctors, 1 dietitian and 1 psychologist) took part in telephone interviews. Interviews lasted between 20 and 55 minutes.

### Themes

The final Template ( Table 3) has four high-level themes. These and their sub-themes illustrated with quotes from the online survey and the interviews are described below.

**Table 3.** Final template.

**1. From fixer to facilitator**		
1.1 The drive to fix	I always felt like I had to fix things	Pauline
1.1.2 The burden of fixing	The burden was like that kind of need to solve	Becky
1.1.3 Letting go of fixing	Moving from trying to fix things	Leanne
1.1.4 Don’t Need to fix	I now believe and understand that it is not important or necessary for me to have the solutions	Iris
1.1.5 Being a rescuer	It’s hard not being a hero	Naveed
1.1.6 Stepping back from fixing	The biggest impact was to be once again reminded about the power of stepping back and moving away from the desire to fix. Without periodic or even constant reflection I think for many of us that go into healthcare - including me - I find I am pulled into that dynamic.	Heather
1.2 Facilitation		
1.2.1 Letting go of control	I’m very good at telling people what to do … Its taught me to give the patient more control in the consultation – (after over 20 years) that’s a big breakthrough for me	Fran
1.2.2 Responsibilities are shared	What’s been really valuable is asking the right questions, to help people to think about what they need to do to solve the issue	Pauline
1.2.3 Not being afraid of silences	The silence part is the biggest thing- not feeling uncomfortable in the silence	Pauline
1.2.4 Letting go of advice	I realise that being diagnosed with diabetes has a huge psychological impact on an individual and when it comes to advice, less is more.	Sue
1.2.4 Awareness of person-centred values	The core conditions were unconscious now they are conscious	Nadia
1.2.5 Communicating empathy, congruence, unconditional positive regard.	Communicating core conditions rather than just feeling them	Nadia
1.2.6 Skills & reflection	The course not only taught me the counselling techniques, but also made me reflect on my practice as a doctor and the impact of my verbal and non-verbal communication	Patsy
1.2.7 Facilitation-letting go of fixing	It just felt really freeing, like literally being able to facilitate rather than to actually teach or educate.	Becky
1.2.8 A framework	It gave me a framework for which I could approach the issues that I was dealing with	Nadia
1.2.9 Facilitated consultations	The course has transformed how I talk to patients	Josh
1.2.10 Clarity of philosophy	Knuston certainly gave me the structure but it also sort of made it okay, acceptable, permission that it’s okay to work this way.	Nadia
**2. Being the way I want to be**		
2.1 Person centred values	I now wear them (core conditions) on my sleeve and I’m quite proud of them …	Nadia
2.2 Accepting of self	It has allowed me to be happier with who I am now	Nadia
2.3 Trying to do this for years	This is probably what I’ve been trying to do for years in my practice … for years.	Becky
2.4 Affirming	Massively confidence building for me because I I’d often felt professionally, that I was out on a limb	Becky
2.5 Improved relatedness	I feel more connected with each patient rather than with the disease	Naveed
2.6 Collaborative v authoritative	Somebody comes into the clinic not monitoring, previously I would have said ‘well you should monitor’	Nadia
2.7 Changing relationships		
2.7.1 With self	It has helped me accept myself as a person who makes mistakes but who is trying to do better	Naveed
2.7.2 With patients	All it needed was a conversation with a non-judgemental attitude, just go in with an open mind and speak to the patient, not bring prejudice into that conversation	Nadia
2.7.3 With colleagues	It has helped me in relationships with other team members that I work with and the fact that I’m more able to listen to their feedback and not be thinking about what I’m going to do next, trying to solve a problem in my head before I’ve even listened to what the problem is.	Becky
2.7.4 With family	In order to listen to them, you need to give them your full attention. I think that’s changed my relationship with my wife especially. I’m listening better than I was before.	Naveed
2.8 Transforming	It felt almost life changing for me.	Becky
2.9 This is how I wanted to be	If I tried, I couldn’t not apply these core conditions. They’ve permeated through every aspect of what I do, every single aspect and that’s exactly the way I want to be.	Nadia
**3. Reflecting on self**		
3.1 Reflecting is a process	Attending the course … made me reflect on my practice as a doctor	Patsy
3.2 Opportunity to reflect	Previously I was approaching my sessions with my patients with a prior agenda. This course helped me reflect upon how unhelpful this is, as it inhibits the patients from finding their own solutions, to explore the problems in their own ways	David
	In my interactions with patients, I feel that I am more sympathetic to their personal barriers to behaviour change and have more patience when it comes to addressing underlying issues. I now understand that simply "laying down the rules" for diabetes management will not work	Aisling
3.3 Accepting oneself/Accepting others	It’s made me more content with myself	Naveed
	It has helped me accept myself as a person who makes mistakes but who is trying to do better	Naveed
3.4 Relationships with people with diabetes	It helped me to see patients in a different light, as individuals, as you and me.	Naveed
3.4.1 With oneself	I'm better at reflecting on my natural ability/downfall, to know what's wrong (with patients) and that I have the solutions …	Fran
3.4.2 With others personally	Even on a personal level with my wife and my kids I’m probably more accepting than before.	Naveed
3.4.3 Teamwork	Realisation that the team are not asking for fixes but to be facilitated to find their own solutions.	Nadia
**4. Personal and professional change**		
4.1 Built confidence	The course increased my confidence in dealing with people with diabetes	Mary
4.2 Life-changing	It felt life-changing for me on a personal level	Becky
4.3 Personal change	On a more personal level, I recognised and understood some of my own feelings and that helped me a great deal in dealing with some of my personal issues.	Patsy
4.4 More relaxed	I have a sense of myself as being more relaxed and easy-going …	James
4.5 Less combative	The temperature of the conversation is a little bit more mutual, a little bit more understanding	James
4.6 Professional change	There’s only me that has changed- the people who come to the clinic haven’t changed	Nadia
4.7 Feeling more effective	I came away feeling more effective	Jenny
4.8 Confidence	I came away with a greater sense of value for the work I do	Jenny
4.9 Transformation	The course was the catalyst that propelled my transformation from a physician who desired to be a good doctor to actually being a good doctor	Nadia
4.10 Personal & professional change	My interaction with people in general changed to a better more effective one	Patsy

**Theme 1 From fixer to facilitator**

Participants described a journey of change from a ‘fixing’ style of consultation, where they used their expertise to dictate solutions to people with diabetes, to a more facilitative style of consultation, where they engaged with the person with diabetes and focused on what was important for them.

James reflects on his experience of the pressures to be a fixer and then the change in his consultations since becoming more facilitative:

*I do think GPs often feel under pressure to come up with answers … Because we can refer as well, we’ve got the whole range of opportunities potentially, to fix things.* *But, in every sense, I think things (consultations) have improved overall. People have got a lot of things off their chest which were not on the initial agenda in the consultation. I think people are setting the agenda a lot more. We end up talking a lot more about their chosen subjects, rather than mine and they’re happy with that.* **James, general practitioner**

Pauline describes her experience of managing a consultation and feeling an urge to fix but then realising the effectiveness of taking a step back and facilitating:

*I think the one thing that I learnt more than anything was to accept that I cannot always "fix things" and that I should stop trying. It is easy to give someone the answer. It is however much better to help them come up with the answer themselves.* **Pauline, diabetes specialist nurse**

**Overcoming a fear of silence**

A feature of ‘fixing’ was a fear of silence and the strong desire to fill that space within a consultation. Several participants talked of coming to understand that silences gave a person with diabetes time to process and reflect.

*What did I take away? I think it was … not having to fear the silence quite so much, I talk a lot, I just do, I talk a lot and sometimes it is, I just cackle along, to fill the gaps, I think it’s a fairly common trait but I do think within a consultation process what I’ve taken away is not being afraid to sit and listen, to paraphrase what they’re saying so that we both understand and not being afraid to let them provide their own answers.* **Jenny, paediatric diabetes specialist nurse**

**Becoming unburdened**

Providing solutions for people with diabetes and finding that they returned with the issues unresolved had been stressful for ‘fixers’. Participants described how they had experienced a weight of responsibility for glycaemic control and diabetes outcomes in individuals with diabetes. ‘Fixers’ tended to blame themselves using words including ‘ineffective’, ‘hopeless’, ‘a failure’ when people under their care did not do well.

*I have often felt that what I was offering as a professional was not eliciting the desired effect. Merely giving information on management and pointing out the obvious like ‘you need to take care of your diabetes’ or ‘take insulin regularly’ was utterly ineffective. Did this mean I was a poor physician? I have always aspired and endeavoured to be a good doctor. A personal sense of failure was prominent.* **Nadia, consultant physician**

Becky describes this solution-focussed way of working as a *‘burden’* and that working in a person-centred way felt *‘like a weight lifted off my shoulders’*:

*The burden was kind of like that need to solve, obviously in a caring profession, that kind of need to find the answer and solve it for someone … it just felt really freeing, like literally being able to facilitate rather than to actually teach or educate, just kind of letting someone see and explore things which they clearly hadn’t explored.* **Becky, diabetes specialist nurse**

The experience of changing from fixer to facilitator deepened most participants’ understandings of person-centred philosophy. Participants reflected on how this philosophy fitted with their beliefs about practice not only in terms of communication but within the environments and systems of care participants work in.

**Theme 2 Being how I want to be**

Participants found that working with a person-centred approach allowed them to feel true to themselves and their personal values.

*I’ve given myself permission to step out and not be the consultant that everybody expects me to be but be the physician that my patients need me to be … as stupid as it sounds, it actually has allowed me to be happier with who I am now.* **Nadia, consultant physician**

Becky describes a sense of relief at how finding the tools helped her to become person-centred. To be able to function as a congruent clinician had a profoundly positive effect on her:

*It’s probably the approach I’ve always wanted to take but this gave me structure … it sort of felt life changing for me. I think I’m more confident because I feel I can genuinely be myself and it feels authentic for me.* **Becky, diabetes specialist nurse**

**Theme 3 Reflecting on self**

Applying person-centred values to people with diabetes also led participants to apply those values to themselves:

*I think the course helps you reflect on yourself and your behaviour … by the fact that you accept the patient as they are, you try to accept yourself as you are. You have more acceptance of who you are and what you do. I think it has made me more content with myself.* **Naveed, middle grade doctor**

Nadia realised she was applying her own values to people with diabetes. As she was able to let go of her own drive to be perfect, she was able to let go of expecting perfection in people with diabetes.

*I sort of realised as … as human beings, as people, it’s okay for us not to be at our absolute best all the time. I gave myself permission to be okay at most things and I did not need to be perfect … that’s been huge … but then the penny dropped … that’s exactly what I’m expecting patients to do every time they walk in, expecting them to be perfect, so unconsciously I’m expecting them to do everything right* **Nadia, consultant physician**

Both Nadia and Naveed described letting go of judgemental attitudes towards people with diabetes and towards themselves.

**Theme 4 Changing personally and professionally**

Naveed described change in the way he would respectfully listen, not only to people with diabetes and work colleagues, but to his family:

*I think the course was more than just something to do in the clinic … . it’s about personal behaviours, about you as a person. I think it starts with you, and when it starts with you as a person you start questioning what you do. Then when you change what you’re doing, you find that it doesn’t only apply to clinical, it applies to your day-to-day life. So if someone is talking to me and I’m doing something else, I stop. Either I ask them to come back later when I have done what I’m doing, or I give them my full attention. That’s from my wife to my colleagues at work. I think that’s just a simple example of giving attention to the other person and respecting them and expecting the same. Now if I speak to someone and they’re not respecting me, I just stop talking until they listen, or I walk away, sometimes.* **Naveed, middle grade doctor**

Nadia reflected how the change in her had affected others:

*The only person that changed in that one year is me, none of my patients have changed, they haven’t been on any courses, but I am seeing such differences in the clinic. The clinic feels good.* **Nadia, consultant physician**

Jenny described a change that spoke of increased confidence, role satisfaction and self-esteem:

*I came away with a greater sense of value for the work that I do … I came away feeling more effective* ***.* Jenny, paediatric diabetes specialist nurse**

James spoke of long-term professional change because of the personal reward he experiences from seeing the improved responsiveness in his patients as a result of working in this way:

*The extent to which I have adopted the learning I think has actually lasted, because I found it very satisfying. It works for me, it’s as good as having a really good new drug. The satisfaction I get from seeing people feeling a little bit better is continuing the motivation for me. It’s not costing me a lot in terms of time. You get snappier at it. There is no reason for it to decline, in my mind. It’s an important part of the way I operate now* **James, general practitioner**

Josh, like other participants, reflected that change became well established over time underpinned by a person-centred approach.

*The course has transformed how I talk to patients. The principles of being equal partners, (and the concept of) self-empowerment are well grounded in the course which has continued to form my practice till now. The skills I picked up were invaluable, and over time, continue to develop and strengthen.* **Josh, consultant physician**

## Discussion

The aim of empowerment education is to facilitate self-empowerment in people with diabetes. Our research shows that there are also positive outcomes for clinicians who move from fixer to facilitator. This is attributed to the experiential nature of the course, through which participants grow relationships based on the philosophy of Rogers’ Person-Centred Approach. ^15^ Participants demonstrated this philosophy using communication skills and a five-step empowerment consultation structure that facilitates reflection and increases self-autonomy in people with diabetes. ^12^ The changes took place deep within the being of the participants who came to know that not only do patients have the capacity to grow and fulfil their potential but so too, do they. Rogers described this active, more congruent state as encompassing the ideal of the ‘fully functioning person’. ^23^

Participants made references to the day-to-day heavy burden of being a ‘fixer’ and needing to find solutions. This is pathognomonic of the Rescuer role on the Drama Triangle described by Karpman. ^24^ The Triangle describes inauthentic relationships in which personal responsibility is lacking. The antithesis to Karpman’s triangle is the Winning Triangle described by Choy ^25^ where authenticity and personal responsibility are key to successful collaborative relationships. Studies have demonstrated clinicians’ solutions alone may be at variance with peoples’ needs ^26^^–^^28^ which can further increase clinicians’ burden. Viewing this vicious circle through the lens of the Drama Triangle it is conjectured that clinicians engaging with patients through the Rescuer role could increase risk of burnout, characterised by emotional exhaustion, depersonalisation, and a sense of failure. ^29^ In contrast, participants described how engaging more congruently, and respectfully responding in the Caring role on the Winning Triangle, changed them from a disease-centred approach (fixer) to a person-centred approach (facilitator). This reduced their sense of personal responsibility for the outcomes of self-management as they learned to respect and accept the ownership of diabetes self-management in the world of their patients.

A recent study observed the responses from clinicians who had self-reported empathic concern and perspective taking traits. ^30^ The authors found clinicians when faced with emotional content during a consultation tended to respond with advice and information rather than with empathic emotional communication. One possible explanation may be the ethos that pervades medicine and associated professions of the traditional medical model of fixing, or, as the authors discuss, empathic clinicians may internalise the patient’s distress and seek to fix it rather than engage with it. Rogerian person-centred theory would hypothesise that such clinicians are not demonstrating empathic understanding of their patients’ emotions but are in an acute state of incongruent awareness, engaging through a Rescuer role on the Drama Triangle. Such a response also has the hallmark of clinician countertransference ^31^ which would explain why they ‘ *received fewer patient expressions of emotions’.*

Becoming a facilitator, reorientated participants from ‘value incongruence’ towards ‘value congruence’. This was defined in earlier research as alignment between individuals’ values and the values held by the organisations they worked within. ^32^ In the present case, this means participants being able to work within their own philosophy rather than the philosophy of traditional training and/or the environment they work in. This is represented by the theme ‘Being how I want to be’, a key characteristic of Rogers’ notion of the ‘fully functioning person’. ^23^ The authors speculate that this, in the long-term, may reduce the risk of stress and burnout.

A strength of this study was the diversity of experienced clinicians who reported personal and professional benefit from the course. Not all clinicians who had participated on the course were contactable partly because details had become out of date over time. As a result, two thirds of participants had undertaken the course in the previous two years. This may be a limitation because non-responders may have felt unchanged by the course, although this is not borne out by the positive evaluations received from clinicians who did not agree to participate. This potential limitation is mitigated by the qualitative nature of the study, which makes no claims to generalisability and is thus less prone to sampling bias.

Future research is required in three areas. First, to investigate which aspects of empowerment education within relationships grounded in person-centred philosophy might mitigate clinician burnout. Secondly, to assess how person-centred philosophy, skills and five-step empowerment model could be introduced more widely into clinical practice. Thirdly to evaluate the impact of this step-change in clinician practice on people with diabetes. In conclusion this study demonstrates that empowerment education has the potential to affect a change in perspective in participants’ approach to people with diabetes. Becoming a facilitator may reduce the personal burden of responsibility for patient self-management outcomes.

## Conclusion

A philosophy based on the Person-Centred Approach, and an empowerment consultation structure that recognizes the patient perspective including psychosocial barriers to diabetes self-management, can transform the facilitation of self-management and behaviour change. It may also benefit professionals in a way which protects against burnout.

## Author contribution statement

**Florence Findlay-White:** Conceptualisation, Methodology, Investigation, Analysis, Writing – Original Draft preparation.

**Tim Dornan:** Conceptualisation, Methodology, Investigation, Analysis, Writing – Original Draft preparation, Supervision.

**Mark Davies:** Conceptualisation, Analysis, Writing – Reviewing and Editing, Supervision.

**Alan Archer:** Conceptualisation, Analysis, Writing – Reviewing and Editing.

**Anne Kilvert:** Conceptualisation, Analysis, Writing – Reviewing and Editing.

**Charles Fox:** Conceptualisation, Analysis, Writing – Reviewing and Editing.

## Data availability

### Underlying data

Since the underlying data are personally very sensitive to participants and the risk of deductive disclosure is unacceptably high, we are unable to publicly share the data. The ethics committee granted ethical approval without questioning this. Readers may apply for access to the data by applying in writing to the first author, by email florencefindlaywhite@gmail.com identifying themselves by name and position held. In keeping with our commitment to preventing inappropriate deductive disclosure, the condition for access is that participants whose data are present in any released material should know who is asking for access to the data and give written approval to release.

### Extended data

Queen's University Belfast: Dataset for “From Fixer to Facilitator”. https://doi.org/10.17034/abba6744-4cb1-45f1-9c5a-2b76588b6d4e ^33^

This project contains the following extended data:

- Letter to participants (pdf and docx)
- Interview guide (pdf and docx)
- Online survey questions (pdf and docx)

Data are available under the terms of the Creative Commons Attribution 4.0 International license (CC-BY 4.0).
