# Supplementary material for: From fixer to facilitator: an interpretative phenomenological study of diabetes person-centred counselling and empowerment-based education
Source: F1000Res. 2023 Nov 9;11:78. Originally published 2022 Jan 21. [Version 2] doi: 10.12688/f1000research.73596.2 (PMC10904953; doi:10.12688/f1000research.73596.2)
Supplement: Peer review history

# Reviewer report: Reviewer response for version 2

**DOI:** 10.5256/f1000research.157703.r243491

**Published:** 2024-02-29

**Authors:** Sarah Lafontaine

**Affiliations:** 1 University of Sherbrooke, Longueuil, Québec,, Canada

Your article is very interesting and relevant. It fits well with the need to develop strategies to facilitate the integration of a person-centred approach into the practice of health professionals.

 Some suggestions and comments:

**Introduction**:

 Is it possible to define a person-centred consultation?

**Methods**:

 It would be interesting to specify the strategy used to optimize the response rate to the survey. Had reminders been sent? How much and how often?

 Have you tried to contact non-respondents in an attempt to understand the reasons for their non-participation (or partial participation in the survey)? It is possible that people who had a more positive experience with the training responded more to the survey than people who had a more negative experience.

**Intervention**:

 It would have been interesting to schematize the training (table 1) in order to quickly identify the content, format and sequence over the 3 days.

**Data collection**:

 Are the telephone interviews fully transcribed (word for word)?

 It would be interesting to add 1 or 2 example(s) of questions from the telephone interview guide directly in the article (in order to avoid the reader having to consult the extended data)

**Results**:

 Did the interviews make it possible to determine whether one of the training activities proved, according to the participants, to be more "effective" in leading to a change in their vision (from fixer to facilitator) and their practice?

 Have divergent results been obtained? (e.g. challenges regarding the application of a person-centered approach)

 Congratulations on your paper and I look forward to reading your future work.

Is the work clearly and accurately presented and does it cite the current literature?

Yes

If applicable, is the statistical analysis and its interpretation appropriate?

Not applicable

Are all the source data underlying the results available to ensure full reproducibility?

No source data required

Is the study design appropriate and is the work technically sound?

Yes

Are the conclusions drawn adequately supported by the results?

Yes

Are sufficient details of methods and analysis provided to allow replication by others?

Yes

Reviewer Expertise:

Qualitative research, diabetes, person-centred approach

I confirm that I have read this submission and believe that I have an appropriate level of expertise to confirm that it is of an acceptable scientific standard, however I have significant reservations, as outlined above.

# Reviewer report: Reviewer response for version 2

**DOI:** 10.5256/f1000research.157703.r221799

**Published:** 2023-11-20

**Authors:** Martha Mitchell Funnell

**Affiliations:** 1 Department of Learning Health Sciences, University of Michigan, Ann Arbor, MI, USA

Thank you for your thoughtful comments and responsiveness to the suggestions.

Is the work clearly and accurately presented and does it cite the current literature?

Yes

If applicable, is the statistical analysis and its interpretation appropriate?

Yes

Are all the source data underlying the results available to ensure full reproducibility?

Partly

Is the study design appropriate and is the work technically sound?

Yes

Are the conclusions drawn adequately supported by the results?

Yes

Are sufficient details of methods and analysis provided to allow replication by others?

Partly

Reviewer Expertise:

Empowerment-based education and training for health professionals and people with diabetes.

I confirm that I have read this submission and believe that I have an appropriate level of expertise to confirm that it is of an acceptable scientific standard.

# Reviewer report: Reviewer response for version 1

**DOI:** 10.5256/f1000research.77257.r175121

**Published:** 2023-06-12

**Authors:** Jennifer Halliday

**Affiliations:** 1 Deakin University, Geelong, Australia

Thankyou to the authors for the opportunity to read and review this interesting paper. It is clear from the quotes that the participants gained benefits from taking part in the course, and that they perceived empowerment education/practice to enhance their consultations.

 I enjoyed reading the quotes and felt the tables were well presented. I thought the authors did a very good job of describing the approach to analysis and reflexivity of the researchers. I felt some other areas of the methods could use some additional detail – for example, Who transcribed the interviews? Could you add a brief description of the questions/topics of the survey and interview (as not all readers will take the time to refer to the extended data documents)? There are useful checklists available via the Equator Network website, such as the COREQ or SRQR which would help the authors to easily identify and address such gaps in the reporting. Many journals now require submission of such checklists along with the manuscript.

 A few minor comments relevant to specific sections:

 Introduction

- “Success depends mainly on individual self-management, including dietary behaviours, medication/insulin management, and monitoring.” - Suggest revising this sentence, as stating that some people with diabetes have 'success' with managing diabetes implies that others 'fail' (potential judgemental undertone, which I'm sure is not the intention of the authors). Perhaps see Speight, J. et al. 2021. Our language matters: Improving communication with and about people with diabetes. A position statement by Diabetes Australia. Diabetes Research and Clinical Practice, 173, p.108655.
- “Course participants said that attending the course not only enhanced their care for people with diabetes but had a ‘transformative’ effect on their clinical practice and their personal lives” – this reads like a result, suggest delete from introduction.

Methods

- “The online survey comprised four demographic questions and four open-ended questions about their experiences during and after the course” – I feel this would fit better in the section about data collection as it describes the survey. Also could you clarify the types of experiences the questions explored?
- “Length of interview was determined by the depth in which a participant was willing or able to discuss the topic.” – I noted the interview schedule stated they would be a maximum of 60 minutes – suggest to add to the main manuscript that you aimed to keep interview under 1 hour.
- “The dataset comprised written responses to the online survey and telephone interviews.” – The transcripts and audio recordings of the telephone interviews are the data (the telephone interview itself is the method) – I suggest amending the sentence.

Results/discussion

- I am curious whether there was any diversity in people’s views or experiences? This has not been explored/reported.
- “In conclusion this study demonstrates that empowerment education has the potential to affect a change in perspective in participants’ approach to people with diabetes. Becoming a facilitator may reduce the personal burden of responsibility for patient self management outcomes.” – is this supposed to be the first sentence of the conclusion? It seems odd that there are two conclusions.

Conclusion

- “It may also benefit professionals in a way which protects against burnout.” - I suggest deleting this from the conclusion. As you say, it would be an interesting area for future research, but your study did not investigate it. I suggest instead to add a sentence around the benefits the participants reported experiencing as a result of taking part in the empowerment education course (e.g. lifting the burden of needing to fix things and affirming person-centred approaches to diabetes care) or similar to what you wrote in the first two sentences of the Abstract conclusion.

Thank you again for inviting me to review this paper, I genuinely enjoyed reading it.

Is the work clearly and accurately presented and does it cite the current literature?

Yes

If applicable, is the statistical analysis and its interpretation appropriate?

Yes

Are all the source data underlying the results available to ensure full reproducibility?

No source data required

Is the study design appropriate and is the work technically sound?

Yes

Are the conclusions drawn adequately supported by the results?

Partly

Are sufficient details of methods and analysis provided to allow replication by others?

Partly

Reviewer Expertise:

Psychological aspects of diabetes

I confirm that I have read this submission and believe that I have an appropriate level of expertise to confirm that it is of an acceptable scientific standard, however I have significant reservations, as outlined above.

## References

[1] : Our language matters: Improving communication with and about people with diabetes. A position statement by Diabetes Australia. *Diabetes Res Clin Pract* .2021;173: 10.1016/j.diabres.2021.108655 108655 10.1016/j.diabres.2021.108655 33422586

# Response

**Published:** 2023-10-31

**Authors:** Florence Findlay-White

**Affiliations:** Queen's University Belfast, UK

**From Fixer to Facilitator. Findlay-White et al. Authors response to reviews**

***Reviewers’ critical comments, tagged by reviewer number***

***Authors’ response, where indicated***

***Revisions***

**General comments**

 R1: Self-empowerment is redundant. We cannot empower our patients. By definition, empowerment is inherent in a self-managed illness

 -We agree

 -One occurrence was a verbatim quote, which should not be changed. We have deleted the word ‘self-‘ in the second occurrence (Discussion, first line)

 R1: the phase "empowerment education" is not clear. We can provide empowerment-based education to patients and teach empowerment-based strategies to both other health professionals and patients.

 -We agree and have used the term empowerment-based throughout.    

 Also, only MDs get "medical" training or education. Professional would be the more accurate description.

 -We used the term ‘medical’ accurately because each occurrence criticises the type of education epitomised by the profession of medicine.

**Introduction**

 R1: While empowerment (especially in the early work) were based on Frier and Rogers, the 5-step model and other strategies are based on self-determination theory and self-discovery educational methods. This should be included for accuracy and to take a more current view of this work. (See: Funnell MM. (2016).

 -We thank R1 for this important point.

 -We have updated our citation of her work to the 2016 article she cites (Reference 12) and cited her point verbatim in the revised manuscript.

 R2: “Success depends mainly on individual self-management, including dietary behaviours, medication/insulin management, and monitoring.” - Suggest revising this sentence, as stating that some people with diabetes have 'success' with managing diabetes implies that others 'fail' (potential judgemental undertone, which I'm sure is not the intention of the authors). Perhaps see Speight, J. et al. 2021. Our language matters: Improving communication with and about people with diabetes. A position statement by Diabetes Australia. Diabetes Research and Clinical Practice, 173, p.108655.

 -Point taken. We don’t think it is necessary to cite the position statement because the point is so self-evidently important.

 -We have replaced the word ‘success’ with the word ‘this’.

 R2: “Course participants said that attending the course not only enhanced their care for people with diabetes but had a ‘transformative’ effect on their clinical practice and their personal lives” – this reads like a result, suggest delete from introduction.

 -We agree that this phrase in Introduction foreshadows the results, but we don’t adhere to rigid application of IMRAD. Making the proposed change would impact the logic of the research because, without giving that important piece of background, there would have been no logic behind researching the phenomenon of ‘transformation’.

**Methods**

**Are sufficient details of methods and analysis provided to allow replication by others?**

 R1: Partly

 R2: Partly: I felt some areas of the methods could use some additional detail

 -We note the agreement between the two reviewers and have listed their specific requests below.

 -See below

**Are all the source data underlying the results available to ensure full reproducibility?**

 R1: Partly

 R2: No source data required

 -We note the difference of opinion between the two reviewers.

 -Since some readers might share R1’s concern, we have added a sentence in the ethical approval section reporting our ethical concerns about making source data openly available. We point out that the article contains a very comprehensive statement of data availability, including the option to ask us for the source data.

 Recruitment

 R1: the response rate was disappointing

 -R1 touches here on an important methodological point. The adjective ‘disappointing’ suggests that the response rate compromised the quality of the research, which assumes that recruitment should be representative of the denominator population. We would have agreed with R1’s epistemological position if this were quantitative research, but this is qualitative research. Opinion is shifting amongst progressive qualitative researchers towards the validity of informative responses as opposed to representative ones.

 -We have commented on this in order to defend the validity of our methods in the context of open publishing, but do not think it would strengthen the article to argue it out in the text. We draw R1’s attention to the sections headed ‘contextual orientation’, ‘reflexive involvement’, and ‘recruitment’, which position the research epistemologically within the relativist/constructivist paradigm.

 R1: It was not clear how the telephone interview participants were chosen, which would be helpful

 -We agree that this was unclear.

 -We have added the word ‘all’ to the final sentence of the final paragraph of ‘Recruitment’.

 R2. Could you add a brief description of the questions/topics of the survey and interview (as not all readers will take the time to refer to the extended data documents)? There are useful checklists available via the Equator Network website, such as the COREQ or SRQR which would help the authors to easily identify and address such gaps in the reporting. Many journals now require submission of such checklists along with the manuscript.

 -We do not share R2’s enthusiasm for checklists, which tend to have an inherently strong positivist bias, and are therefore at odds with our constructivist position.

 -We have included brief precis of the survey and interview in the text, as R2 requested.

 Data collection

 R2. “The online survey comprised four demographic questions and four open-ended questions about their experiences during and after the course” – I feel this would fit better in the section about data collection as it describes the survey. Also could you clarify the types of experiences the questions explored?

 -Our response to R2’s first point is the same as our response, above, to her critique of the final paragraph of Introduction. In reality, research is an iterative process. We could not have described recruitment to the Telephone interviews without describing the content of the survey instrument so taking up this suggestion would replace one problem with another problem.

 -We have not made the suggested change for the sake of readability and coherence.

 -Please see response immediately above referring R2 to the place in the revised manuscript where we clarified the survey procedure.

 R2: “Length of interview was determined by the depth in which a participant was willing or able to discuss the topic.” – I noted the interview schedule stated they would be a maximum of 60 minutes – suggest to add to the main manuscript that you aimed to keep interview under 1 hour.

 -We take this point.

 -The relevant sentence in ‘data collection’ now reads: ‘Interviews lasted up to 1 hour, their exact length being determined …..’

 R2: “The dataset comprised written responses to the online survey and telephone interviews.” – The transcripts and audio recordings of the telephone interviews are the data (the telephone interview itself is the method) – I suggest amending the sentence.

 -We have amended the first sentence of Analytical procedures to: ‘ …. and transcripts of telephone interviews.’

 Analytical procedures

 R2: Who transcribed the interviews?

 -We have added the phrase: ‘…which were transcribed by a commercial bureau.’

 R2: I thought the authors did a very good job of describing the approach to analysis and reflexivity of the researchers.

 -We appreciate this positive comment.

**Results**

 R2: I am curious whether there was any diversity in people’s views or experiences? This has not been explored/reported.

 -We have added the sentence: ‘There was a high level of consistency between participants in their experiences; verbatim quotations, below, illustrate some personal variations.’

**Discussion**

 Are the conclusions drawn adequately supported by the results?

 R1: Yes

 R2: Partly

 Please see the response below, in which we have revised the conclusions into a single, more concise statement.

 R2: “In conclusion this study demonstrates that empowerment education has the potential to affect a change in perspective in participants’ approach to people with diabetes. Becoming a facilitator may reduce the personal burden of responsibility for patient self management outcomes.” – is this supposed to be the first sentence of the conclusion? It seems odd that there are two conclusions.

 R2: “It may also benefit professionals in a way which protects against burnout.” - I suggest deleting this from the conclusion. As you say, it would be an interesting area for future research, but your study did not investigate it. I suggest instead to add a sentence around the benefits the participants reported experiencing as a result of taking part in the empowerment education course (e.g. lifting the burden of needing to fix things and affirming person-centred approaches to diabetes care) or similar to what you wrote in the first two sentences of the Abstract conclusion.

 -Point well taken.

 -We have condensed those two paragraphs into a single conclusions section, containing the best of both the previous ones.

# Reviewer report: Reviewer response for version 1

**DOI:** 10.5256/f1000research.77257.r120710

**Published:** 2022-02-15

**Authors:** Martha Mitchell Funnell

**Affiliations:** 1 Department of Learning Health Sciences, University of Michigan, Ann Arbor, MI, USA

This well-written and well-done manuscript adds to the understanding of the necessity and value of health care training in patient-centered, empowerment-based strategies. The quotes were interesting and supported the conclusions. Although the response rate was disappointing, the results are interesting and will be helpful for future offerings.

 Minor comments:

- It was not clear how the telephone interview participants were chosen, which would be helpful.
- Language: Self-empowerment is redundant. We cannot empower our patients. By definition, empowerment is inherent in a self-managed illness. In the same token, the phase "empowerment education" is not clear. We can provide empowerment-based education to patients and teach empowerment-based strategies to both other health professionals and patients. Also, only MDs get "medical" training or education. Professional would be the more accurate description.
- While empowerment (especially in the early work) were based on Frier and Rogers, the 5-step model and other strategies are based on self-determination theory and self-discovery educational methods. This should be included for accuracy and to take a more current view of this work. (See: Funnell MM. (2016). ^1^ 

Is the work clearly and accurately presented and does it cite the current literature?

Yes

If applicable, is the statistical analysis and its interpretation appropriate?

Yes

Are all the source data underlying the results available to ensure full reproducibility?

Partly

Is the study design appropriate and is the work technically sound?

Yes

Are the conclusions drawn adequately supported by the results?

Yes

Are sufficient details of methods and analysis provided to allow replication by others?

Partly

Reviewer Expertise:

Empowerment-based education and training for health professionals and people with diabetes.

I confirm that I have read this submission and believe that I have an appropriate level of expertise to confirm that it is of an acceptable scientific standard, however I have significant reservations, as outlined above.

## References

[1] : Patient empowerment: What does it really mean?. *Patient Education and Counseling* .2016;99(12) : 10.1016/j.pec.2016.10.010 1921-1922 10.1016/j.pec.2016.10.010 27871730

# Response

**Published:** 2022-02-15

**Authors:** Charles Fox

**Affiliations:** Diabetes Counselling Course, UK

Thank you Marti. I feel privileged that you took time to review our article. We will take your comments seriously and work out how to respond to your suggestions. Thanks for the 2016 editorial.

 Yours, Charles Fox
